# Prevalence and prognostic impact of BRAF V600E mutation and CDKN2A deletion in pediatric high-grade glioma

**DOI:** 10.3389/fonc.2025.1537268

**Published:** 2025-08-08

**Authors:** Soha Karim, Amal Mosaab, Hala Taha, Iman Naguib, Amal Refaat, Eslam Maher, Mohamed Saad Zaghloul, Mohamed El-Beltagy, Hanafy Hafez, Mohamed Kamal, Sherine Salem, Mark W. Kieran, Ahmed El-Hemaly, Alaa El-Haddad

**Affiliations:** ^1^ Pediatric Oncology Department, Children’s Cancer Hospital Egypt, Cairo, Egypt; ^2^ Tumor Biology Research Program, Basic Research Unit, Research Department, Children’s Cancer Hospital Egypt, Cairo, Egypt; ^3^ Department of Pathology, Children’s Cancer Hospital Egypt, Cairo, Egypt; ^4^ Department of Pathology, National Cancer Institute, Cairo University, Cairo, Egypt; ^5^ Department of Radio Diagnosis, Children’s Cancer Hospital Egypt, Cairo, Egypt; ^6^ Department of Radio Diagnosis, National Cancer Institute, Cairo University, Cairo, Egypt; ^7^ Department of Surgery and Cancer, Faculty of Medicine, Imperial College London, London, United Kingdom; ^8^ Department of Clinical Research, Children’s Cancer Hospital Egypt, Cairo, Egypt; ^9^ Department of Radiation Oncology, National Cancer Institute, Cairo University, Cairo, Egypt; ^10^ Department of Radiation Oncology, Children’s Cancer Hospital Egypt, Cairo, Egypt; ^11^ Department of Neurosurgery, Kasr Al-Ainy School of Medicine, Cairo University, Cairo, Egypt; ^12^ Department of Neurosurgery, Children’s Cancer Hospital Egypt, Cairo, Egypt; ^13^ Department of Pediatric Oncology, National Cancer Institute, Cairo University, Cairo, Egypt; ^14^ Molecular Pathology Lab, Children’s Cancer Hospital Egypt, Cairo, Egypt; ^15^ Department of Clinical Pathology, Children’s Cancer Hospital Egypt, Cairo, Egypt; ^16^ Department of Clinical pathology, National Cancer Institute, Cairo University, Cairo, Egypt

**Keywords:** pediatric, high grade, glioma, BRAF, CDKN2A, prevalence, prognostic factors

## Abstract

**Background and aim:**

Pediatric high-grade glioma (pHGG) is a rare and challenging disease with dismal outcomes. Identifying prognostic markers and targeted therapeutic avenues is crucial.

**Methods:**

We conducted a retrospective study involving 130 pediatric patients with HGG treated at the Children’s Cancer Hospital Egypt between July 2007 and December 2018. Demographic, clinical, and molecular data were collected, and BRAF V600E mutation and CDKN2A deletion status were assessed.

**Results:**

Tumor site, and extent of resection significantly influenced outcomes (P value 0.001 for both). Out of 130 patients, 128 underwent BRAF analysis and 7% harbored the BRAF V600E mutation (n:9) and 129 patients underwent CDKN2A analysis. Of those, 78 patients (60%) had CDKN2A deletion, 18 patients had homozygous CDKN2A deletions, and the remaining 60 patients had heterozygous deletions. BRAF V600E mutation and CDKN2A status (deleted vs normal) did not affect the outcome (p value 0.46 and 0.23, respectively). The 3 years Overall survival and Progression free survival rates were 26.2% and 23.4%, respectively.

**Conclusion:**

Neither BRAF V600E mutation nor CDKN2A deletion status significantly impacted this cohort’s progression-free survival (PFS). Patients with these mutations now have access to new targeted BRAF V600E inhibitors, which may improve their outcomes.

## Introduction

Central nervous system (CNS) tumors are childhood’s most common solid neoplasms. Among these, approximately 50% are gliomas. According to the World Health Organization (WHO), gliomas are histopathologically classified into low-grade gliomas (LGG) (grade 1-2) and high-grade gliomas (HGG) (grade 3–4) (1). Pediatric HGGs (pHGGs) are rare, accounting for one-third of pediatric gliomas. Several prognostic factors have been identified for the outcome of pHGG, including age, histologic subtype, tumor location, extent of surgical resection, molecular subtyping, and performance status (2).

Treatment of pHGGs includes maximal safe surgical resection, focal radiation therapy, consideration of chemotherapy, and more recently targeted therapy. However, despite maximal combined modality therapies, the outcome is still dismal, with a survival rate of <20%. Thus, there is a need for novel, precision medical approaches, including biomarker research. Molecular profiling has become a crucial step in the diagnosis, prognosis, and treatment of pediatric brain tumors, especially with the increasing availability of novel targeted therapies (3). In recent years, there has been growing interest in investigating the genetic and molecular profiles of pHGGs. Previous studies have identified specific genetic mutations, deletions, insertions, fusions, and amplifications common in pHGGs including H3.3 or H3.1 K27M, H3.3 G34, EGFR, PTEN, TERT, FGFR, IDH, and BRAF (4). Age and gene mutation are independent prognostic factors in pediatric HGG. For example, infantile HGG (iHGG) has been identified as a distinct entity by Clarke et al. reporting on over 130 iHGG. In addition to BRAF, targetable changes were found in most cases including ALK, NTRK1/2/3, ROS1, and MET, some of which correlated with a significantly improved outcome (5).

Studies have identified the extent of surgical resection of pHGG as an independent factor for improved survival, particularly in the context of a gross total resection (6). Histologic grade affects prognosis as well. Patients with low-grade gliomas have an excellent prognosis while those with high-grade tumors, especially those with grade 4 histology or diffuse pontine location, will most likely succumb to disease (7).

B-Raf is a member of the Raf kinase family and a critical component of the MAPK pathway, which is essential for transmitting growth signals to the nucleus. Aberrations in BRAF are present in both low- and high-grade gliomas resulting in constitutive activation of the kinase. The most frequent BRAF mutation is BRAF V600E, which leads to uncontrolled cell proliferation and cancer (8). Previous pediatric studies reported that the incidence of BRAF V600E mutation ranged from 10% up to 20% in pHGGs with a higher incidence in pleomorphic xanthoastrocytoma and gangliogliomas (9).

While the BRAF V600E mutation is sufficient to transform cells, mutations in other pathways may play a role in the overall outcome of this patient population. For example, alterations in the tumor suppressor gene CDKN2A may provide a secondary hit that permits the expression of more malignant behavior and the evasion of cell cycle regulation (3). Numerous studies have identified the negative prognostic impact of homozygous CDKN2A deletion in adult glioblastoma and this is now incorporated into the WHO classification of grade 4 glioma. The significance of heterozygous deletion of CDKN2A in the prognosis of adult gliomas remains less clear with contradictory results (10). The incidence of homozygous CDKN2A deletion in pediatric high-grade gliomas has been reported to range from 18 -40% (11, 12). Unlike their adult counterparts, pediatric HGG with homozygous CDKN2A deletions are often detected in the presence of BRAF V600 point mutations (12). Like adults, patients with homozygous deletions appear to have a worse prognosis when compared to those with wild type CDKN2A, with heterozygote deletion falling in between (13).

The prognostic significance of BRAF V600E mutations in pHGG appears to be much better than other non-BRAF V600E molecular groups. A study by Mackay et al. reported that patients with BRAF V600E mutant pHGGs had a significantly better overall survival rate than those without this mutation, with a 2-year survival of 67% (14).

Several clinical trials investigating the use of BRAF inhibitors (BRAFi) with or without MEK inhibitors (MEKi) in children with pHGG have been undertaken (9, 15). Nobre et al. published the findings of a retrospective multi-institutional assessment of 11 pHGG patients receiving BRAF V600E inhibitor treatment with an observed response rate of 36%, a PFS at one year of 27% although all but one patient had progressive disease by 18 months (16).

In contrast to single-agent BRAF V600E inhibition, recent findings from a phase II clinical trial (NCT02684058) reported that the combination of dabrafenib and trametinib treatment led to a 56% response rate and 66% clinical benefit rate in children with recurrent or refractory pHGG (9), and this study supported the recent FDA approval of this combination in pediatric patients.

The current study aimed to estimate the prevalence of BRAF V600E and CDKN2A in pediatric patients with pHGG and assess their prognostic impact on disease outcomes.

## Patient cohort

This retrospective study included 130 pediatric patients diagnosed with HGGs, excluding DIPG patients, treated at the Children’s Cancer Hospital Egypt (CCHE-57357) between July 2007 and December 2018. Patients who were <18 years of age at the time of diagnosis with histopathologically confirmed HGG tumors were included. The institutional review board approved the study. The study was waived from obtaining informed consent due to its retrospective nature. The following data were collected from the patient’s medical electronic files: age, gender, primary tumor site, the extent of surgical excision, pathological subtype, radiotherapy field and doses, chemotherapy received, and the radiological response according to RANO-HGG criteria (**Supplementary Table 1**) (17). Pathological specimens were centrally reviewed according to WHO 2021 criteria by a single neuropathologist. Those with adequate DNA were tested for BRAF V600E using Sanger sequencing and CDKN2A using real-time PCR.

### Treatment regimens

All patients underwent surgical intervention (near or gross total resection, subtotal resection, or biopsy). Gross total resection (GTR) was defined as the removal of 95% or more of the tumor, subtotal resection (STR) (10%–95% resection), and biopsy (<10% resection) (17). Patients received focal radiotherapy for localized tumors, while those with disseminated or multifocal disease received craniospinal or whole cranium on a palliative basis. Chemotherapy regimens (treated ‘as per’) include COG protocol (CCG-945 PCV), consisting of oral etoposide concomitant with radiation followed by a maintenance phase of eight cycles (procarbazine, CCNU, and vincristine) administered four weeks after the end of radiation. The details of the chemotherapy regimens and doses are provided in **Supplementary Figure 1**.

Infants under one-year-old were treated ‘as per’ the Baby POG protocol (POG 9233/34). This regimen includes 73 weeks of chemotherapy (vincristine and cyclophosphamide alternated with cisplatin and etoposide), followed by radiation according to a multidisciplinary team decision, as shown in **Supplementary Figure 2**.

The field of radiotherapy used was focal with a median dose of 5020 cGy (range 2880cGy-6000cGy), the extreme lower dose of radiotherapy was administered on a palliative basis. Temozolomide was used concomitantly with radiation in a small number of patients.

### Identification of BRAF V600E using Sanger sequencing

DNA was extracted from formalin-fixed, paraffin-embedded (FFPE) tissue using QIAamp DNA FFPE Tissue Kit (Qiagen). In brief, five scrolls, each 5 µm thick, were deparaffinized by xylene and the extraction steps were performed according to the manufacturer’s protocol. Extracted DNA was quantified using a NanoDrop spectrophotometer. PCR was conducted using Thermo DreamTaq Green PCR Master Mix (2X) (ThermoFisher Scientific™). Template DNA was amplified using *BRAF* primers designed to cover the region encoding the V600E mutation (**Supplementary Table 2**). Primers were used at a dilution of 10 pmol/μl, and genomic DNA was added to the reaction at a final concentration of 300–500 ng. PCR amplification was performed using a program that has been shown to amplify the product optimally as follows: initial 10 min of denaturation at 95°C then 40 repeated cycles of the following steps—1 min at 95°C for denaturation, 1 min at 53°C for annealing, 1 min at 72°C for elongation, and finally, 10 min at 72°C for a final elongation step. The product was stored at 4°C (**Supplementary Table 3**). Amplified products were purified using the GeneJET PCR Purification Kit (ThermoFisher Scientific™). PCR products were sequenced in both directions using the BigDye Terminator, version 3.1, Cycle Sequencing Kit (Applied Biosystems). Sanger sequencing was performed using a 3500 Dx Series genetic analyzer (Applied Biosystems, Foster City, CA, USA). DNA sequence electropherogram analysis was conducted using Snap Gene software (from GSL Biotech; available at snapgene.com).

### Analysis of CDKN2A deletion by quantitative real-time PCR

A real-time multiplex QPCR application was developed to measure homozygous deletion in the CDKN2A gene, and CDKN2A amplification was compared with simultaneous amplification of an endogenous reference gene (RNaseP). The method was evaluated in 10 positive reference samples containing a homozygous deletion of CDKN2A. A ready-made TaqMan CNV assay was used along with Applied Biosystems TaqMan universal PCR master mix according to manufacture protocol. PCR reactions were performed in 25 µl volumes containing 20 ng of DNA, 12.5 µl master mix, 1 µl CDKN2A assay mix, and 1 µl RNaseP assay mix. The reaction volume was completed to 25 µl using nuclease-free water. PCR conditions were as follows: an initial 2 min 50°C incubation followed by 95°C for 10 min, and then 40 cycles of 95°C for 15 s and 60°C for 1 min. Samples were analyzed in triplicate; a negative and a positive control was included in each run. *Real-Time QPCR Analysis*. The test parameter, threshold cycle (Ct) value of 9, generated by the BioRad cfx96, was analyzed and then exported to Excel, where equations from the standard curve were generated. The cutoffs were calculated as 0 for homozygous deletion and 0.5 for heterozygous deletion, and ≥1.5 was considered duplication.

### Statistical analysis

Overall survival (OS) was defined as the time from diagnosis to death, and progression-free survival (PFS) was the time from diagnosis to recurrence, progression, or death. Survival probabilities were computed using the Kaplan–Meier estimator function and 95% confidence intervals were calculated using the log method.

The inequality of survival curves was tested using the two-sided log-rank test. Cox regression was fitted with PFS as the primary endpoint. The interaction between CDKN2A deletion and BRAF V600E mutation was examined with Wald and Likelihood Ratio tests. All analyses were performed using R v4.1.0 with the package survival (v3.2-11) and survminer (v0.4.9) (18, 19).

## Results

One hundred thirty patients were included with a male-to-female ratio of 1.09:1. The median age of the entire cohort was 8.3 years (range: 0.5–16.8 years). Fourteen patients (14/130; 10.7%) were younger than three years, while 116 patients (89.2%) were three years or above.

Seventy-seven patients had cortical tumors, while 53 patients had midline tumors. No patients presented with metastatic disease. At the time of progression, metastatic disease was documented in twenty-two patients. GTR was achieved in 53 patients, STR in 29, and biopsy was performed in 45 patients. Three patients did not have surgical details. Furthermore, 57 (43.8%) patients had grade 3 tumors, whereas grade 4 histology was identified in 73 (56.2%) patients. Ten patients had anaplastic pleomorphic xanthoastrocytoma, and two patients had anaplastic ganglioglioma (**Table 1**).

**Table 1:** Initial patient characteristics.

Patient Characteristics		Number (130)	Percentage (%)
Age	1 to less than 3 years old	10	7.6
	≥ 3 years old	116	89.2
	<1 year old	4	3%
Gender	Male	68	52.3
	Female	62	47.7
Pathology	Grade Ill	57	43.8
	Grade IV	73	56.2
Pathological subtype	Anaplastic ganglioglioma	2	1.5
	Anaplastic PXA	10	7.7
	Anaplastic astrocytoma	23	17.6
	Anaplastic astroblastoma	1	0.7
	GBM	66	50.7
	Gliomatosis cerebri	1	0.7
	Gliosarcoma	5	3.8
	HGG	22	16.9
Primary site	Midline	53	40.7
	Cortical	77	59.2
Extent of surgical excision	GTR/NTR	53	40.8
	STR/biopsy	74	56.9
	No available data	3	
Radiological response at end of treatment (n=114)	CR	18	13.4
	PR	4	3
	PD	88	67.6
	SD	4	3
BRAF V600E	Wild type	119	91.5
	Mutant	9	6.9
	No result (poor quality of DNA)	2	1.5
CDKN2A	Normal	51	39.2
	Homozygous deletion	18	13.8
	Heterozygous deletion	60	46.15
	No result	1	0.8

GTR, gross total resection; NTR, near total resection; STR, subtotal resection; CR, complete remission; PR, partial remission; PD, progressive disease; SD, stable disease.

A total of 121 patients received radiotherapy. Two patients achieved complete remission after radiation therapy and chemotherapy. Four patients were treated as per the Baby POG protocol (POG 9233/34): two of them did not receive radiotherapy and achieved complete remission by surgery and chemotherapy.

**Figure 1:**
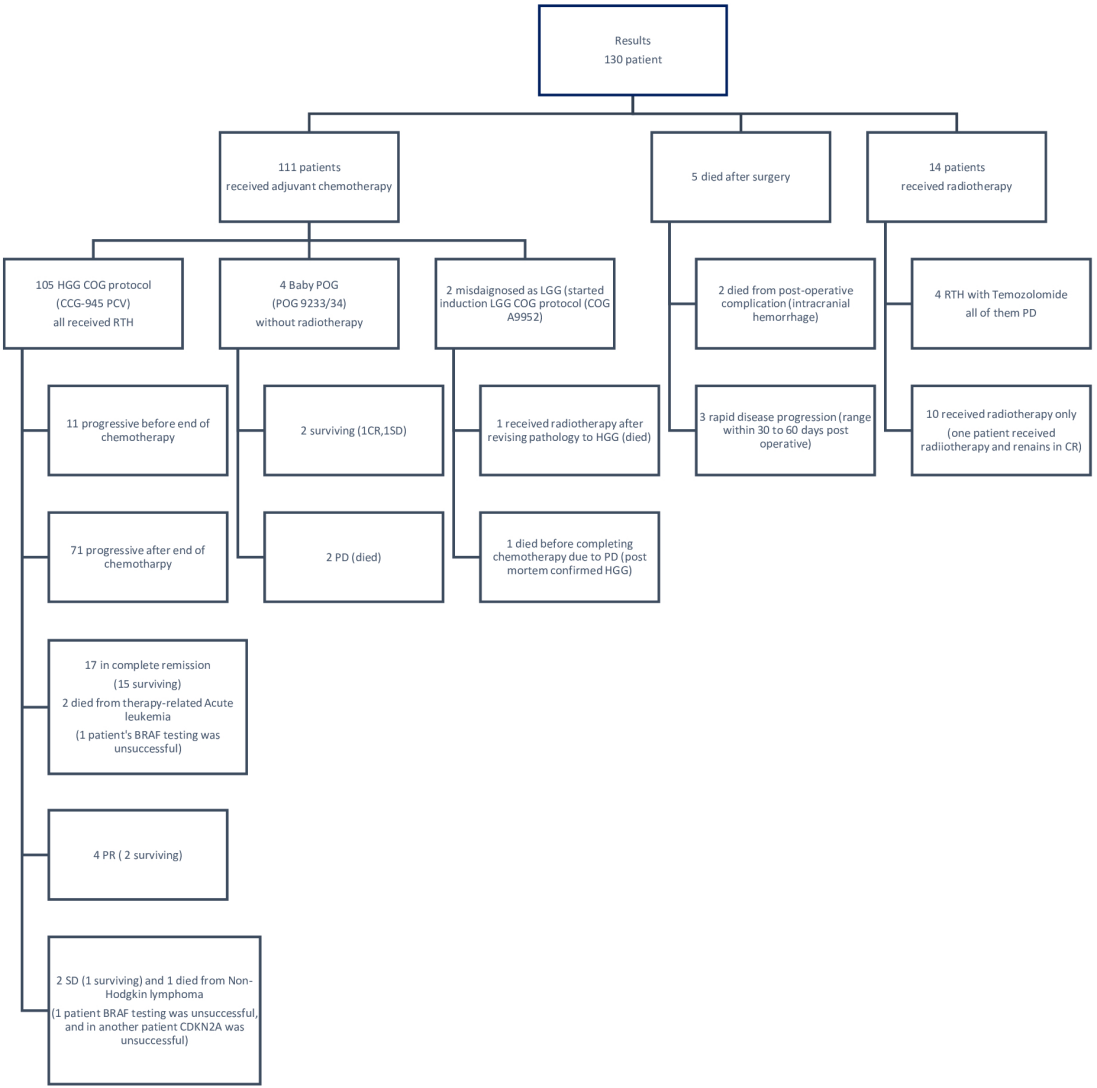
Flowchart shows summary of clinical data of treatment and outcome of all patients.

Ten patients received adjuvant radiotherapy without chemotherapy. Four patients received radiotherapy concomitant with Temozolamide, and five patients died shortly after surgery (two died from intracranial hemorrhage and three died from rapid disease progression before starting treatment). The treatments and outcomes of all patients are summarized in **Figure 1**.

### BRAF status

The BRAF V600E mutation was detected in nine (9/128) (7.0%) patients. Six (6/9) patients also had concurrent CDKN2A deletion: three of them had homozygous deletion and three had heterozygous deletion. Two (2/130) (1.5%) patients were excluded due to poor DNA quality.

The median age of patients with BRAF V600E tumors (nine patients) was 13.5 years (range: 4–16). Seven patients had cortical tumors, while two patients had midline tumors. Five patients had grade 4 tumors and four had grade 3 tumors. One patient achieved complete remission after radiotherapy. At the end of therapy, five (5/9) (55.5%) patients were in complete remission, and partial remission was observed in four patients (44.4%). All patients progressed except one patient with PXA who had both BRAF mutation and heterozygous CDKN2A deletion and remains in complete remission (**Table 2**).

**Table 2:** Clinical characteristics correlated with BRAF V600E status.

Characteristics (all patients = 128)		Wild type Number (n=119)	Percentage	Mutant Number (n=9)	Percentage
Age, years (Median)		8.2	93	13.5	7
Gender	Male	64	50	4	3.1
	Female	55	42.9	5	3.9
Site	Cortical	69	53.9	7	5.4
	Midline	50	39	2	1.5
Extent of surgical excision	GTR/NTR	47	36.7	5	3.9
	STR/Biopsy	70	54.6	4	3.1
	unknown	2	1.5	0	
Pathological grade	Grade Ill	51	39.8	4	3.1
	Grade IV	68	53	5	3.9
Response At end of therapy	CR	16	12.5	1	0.7
	PR	5	3.9	0	0
	SD	4	3.1	0	0
	PD	94	73.4	8	6.2
CDKN2A status	Normal	48	37.5	3	2.3
	Homozygous Deletion	14	10.9	3	2.3
	Heterozygous Deletion	56	43.7	3	2.3
	No result	1	0.7		

### CDKN2A status

A total of 18 patients exhibited homozygous CDKN2A deletions, 60 patients had heterozygous deletions, and 51 had no abnormality. The median age of those with CDKN2A deletions was 8.3 years (range: 0.5–16.8 years). In the homozygous group, 13 patients had cortical tumors, and five had midline tumors. In the heterozygous deletion group, 36 patients had cortical tumors, and 24 had midline tumors (**Table 3**).

**Table 3:** Clinical characteristics correlated with CDKN2A status.

Characteristics (n=129)		Normal (n=51)	Homozygous deleted (n=18)	Heterozygous deleted (n=60)
Age, years(Median)		8.3	8.3	8.3
Gender	Male	27	9	32
	Female	24	9	28
Site	Cortical	28	13	36
	Midline	23	5	24
Extent of surgical excision	GTR/NTR	22	11	25
	STR/Biopsy	29	6	33
	unknown	0	1	2
Pathological grade	Grade Ill	25	6	26
	Grade IV	26	12	34
Response at end of treatment	CR	6	4	8
	PR	1	2	2
	SD	1	1	2
	PD	43	11	48
BRAF V600 E	Wild	48	14	56
	Mutant	3	3	3
	No enough DNA for BRAF		1	1

**Figure 2:**
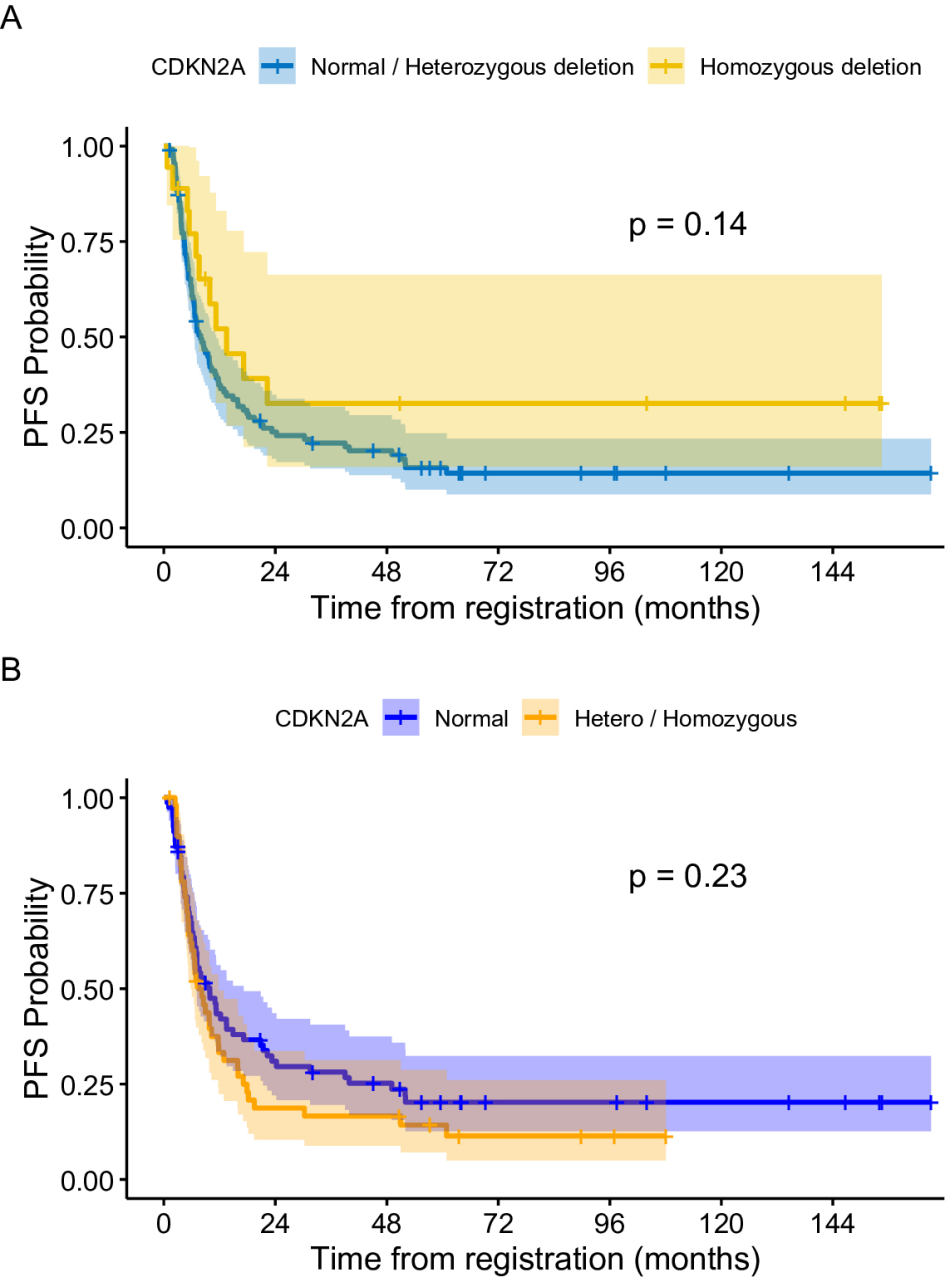
**(A)** Progression free survival of patients based on CDKN2A status (Normal + heterozygous CDKN2A vs homozygous deletion. **(B)** Progression free survival of patients based on CDKN2A status (Normal versus hetero + homozygous deletion.

At the end of treatment, complete remission was observed in four (4/18; 22.2%) patients with homozygous deletion and eight (8/60; 13.3%) with heterozygous deletion. Partial remission was observed in two patients in the heterozygous group and two in the homozygous group. One patient with homozygous deletion and two in the heterozygous group showed stable disease. In total, 11 patients with homozygous deletion and 48 with heterozygous deletion showed progression (**Figure 2**).

### Prognostic factors

Age approached but did not reach statistical significance in terms of PFS, with a three-year PFS rate for patients aged less than 3 of 50% (95% CI: 29.6–84.4) compared to 20% (95% CI: 13.7–29.1) for those three years and above (p = 0.07). The tumor site significantly affected the outcome. Cortical and midline tumors had three-year PFS of 33.5% and 8.9%, respectively (p < 0.001).

The extent of resection also had an impact on PFS. The three-year PFS for the GTR and non-GTR group was 42.1% (95% CI: 30.6%–58%) and 10.5% (95% CI: 5.2%–21%), respectively (p < 0.001).

Regarding the radiological response at the end of therapy based on RANO-HGG, patients who achieved CR, PR or SD had a three-year PFS of 100%, while those with PD had a 3-year PFS of 8% (p <0.001) (**Table 4**). By multivariable analysis, we found that tumor site, extent of resection and pathological grading have significant impact on survival (**Table 5**).

**Table 4:** Univariable progression-free survival (PFS) analyses for key clinical and molecular characteristics.

	Variable	number	3yr PFS (95% CI)	HR (95% CI)	P value
All cases		130	23.4 (17-32.2)	**-**	**-**
Age group	Less than 3	14	50 (29.6-84.4)	1	0.07
	More than 3	116	20 (13.7-29.1)	1.95 (0.94-4.03)	
Gender	Female	62	22.8 (14.2-36.6)	1	0.62
	Male	68	24 (15.5-37)	0.91 (0.62-1.34)	
Tumor site	Cortical	73	33.5 (24.3-46.2)	1	0.001
	Midline	57	8.9 (3.3-21.1)	2.18 (1.47-3.24)	
Extent of resection	GTR/NTR	53	42.1 (30.6-58)	1	< 0.001
	STR/Biopsy	74	10.5 (5.2-21)	2.19 (1.45-3.33)	
Pathological grade	III	57	33.6 (23-49)	1	0.03
	IV	73	15.8 (9.2-27.1)	1.59 (1.06-2.38)	
Radiological response	CR	18	100% (NA)		< 0.001
	PR	4	100% (NA)		
	SD	4	100% (NA)		
	PD	88	8% (3.9 to 16.2%)		
BRAF V600E	Wild type	119	23.9 (17.2-33.3)	1	0.46
	Mutant	9	11.1 (1.8-70.5)	1.31 (0.64-2.71)	
CDKN2A	Normal	51	16.6 (8.9-31.3)	1	0.23
	Deleted	78	28.2 (19.6-40.6)	0.79 (0.53-1.17)	
CDKN2A deletion	No deletion	51	16.6 (8.9-31.3)	1	
	Homozygous	18	32.6 (16-66.3)	0.58 (0.30-1.12)	0.10
	Heterozygous	60	26.8 (17.5-41)	0.86 (0.57-1.3)	0.47

**Table 5:** Adjusted multivariable analysis for the prognostic factors affecting progression-free survival (PFS).

Variable		HR (95% CI)	P
Age group	Less than 3	1	0.18
	More than 3	1.68 (0.79-3.56)	
Tumor site	Cortical	1	0.01
	Midline	1.92 (1.22-3.04)	
Extent of resection	GTR/NTR	1	0.01
	STR/Biopsy	1.80 (1.15-2.83)	
Pathological grade	III	1	< 0.001
	IV	2.09 (1.37-3.21)	

### Survival analysis

**Figure 3:**
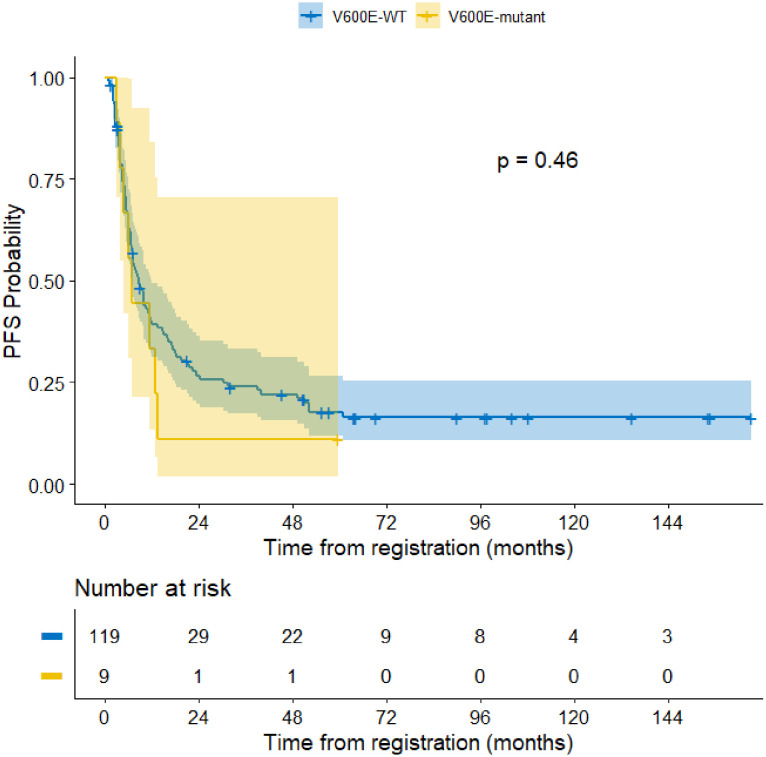
Progression free survival of BRAF V600E mutant cases compared to wild type.

The median follow-up period of the entire cohort was 7.5 years with three-year PFS and OS of 23.4% (95% CI: 17%–32.2%) and 26.2% (95% CI% 19.6–35.1), respectively. At the end of treatment, 18 patients were in complete remission, four patients achieved partial response, four patients had stable disease, and 88 patients had progressive disease. Moreover, 11 patients died of progressive disease before the end of chemotherapy. Patients with BRAF V600E mutation had a 3–year PFS of 11.1% compared to 23.9% for those with wild-type BRAF (p =0.46) (**Figure 3**). The status of CDKN2A did not have a significant impact on survival as the three-year PFS was 16.6% for patients with normal CDKN2A status, 32.6% for those with homozygous deletion, and 26.8% for the heterozygous group (p= 0.14) (**Figure 2**).

An assessment of the impact on survival of BRAF V600E mutational status in the context of CDKN2A deletions and tumor location was performed. There was no significant difference in survival between CDKN2A status in patients with midline tumors. The 3-year PFS for normal, heterozygous, and homozygous deletion of CDKN2A was 10.1% (95% CI:2.7 to 37.3), 8.3% (95% CI 2.2 to 31.4) and 0% respectively (p = 0.7). For cortical tumors, the 3–year PFS of normal, heterozygous and homozygous deletion was 21.4% (95% CI 10.5 to 43.6), 39.8% (95% CI 26.4 to 60.0) and 42.7% (95% CI:22.2 to 82.3), respectively (p = 0.2). An assessment of survival differences based on BRAF mutational status also failed to detect a difference with 3-year PFS of 34.6% (95% CI: 24.8 to 48.1) and 14.3% (95%: CI 2.3 to 87.7) for wild type and mutant tumors, respectively (p = 0.4). A similar result was identified for midline tumors, with a 3-year PFS of 8.8% (95% CI: 3.5 to 22.4) and 0% for wild and mutant types, respectively (p = 0.4).

**Figure 4:**
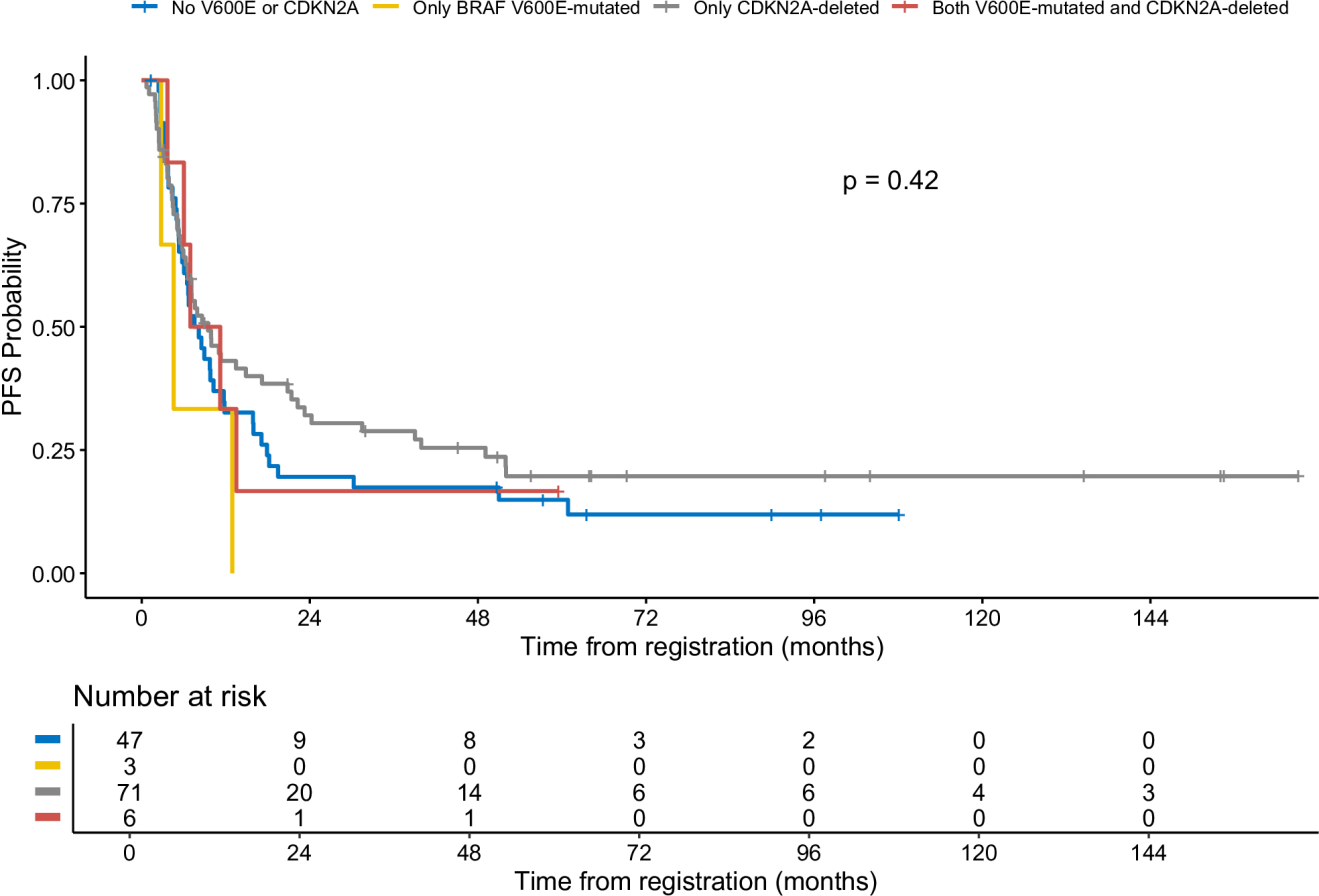
Progression-free survival of patient with different status of BRAF and CDKN2A.

**Figure 5:**
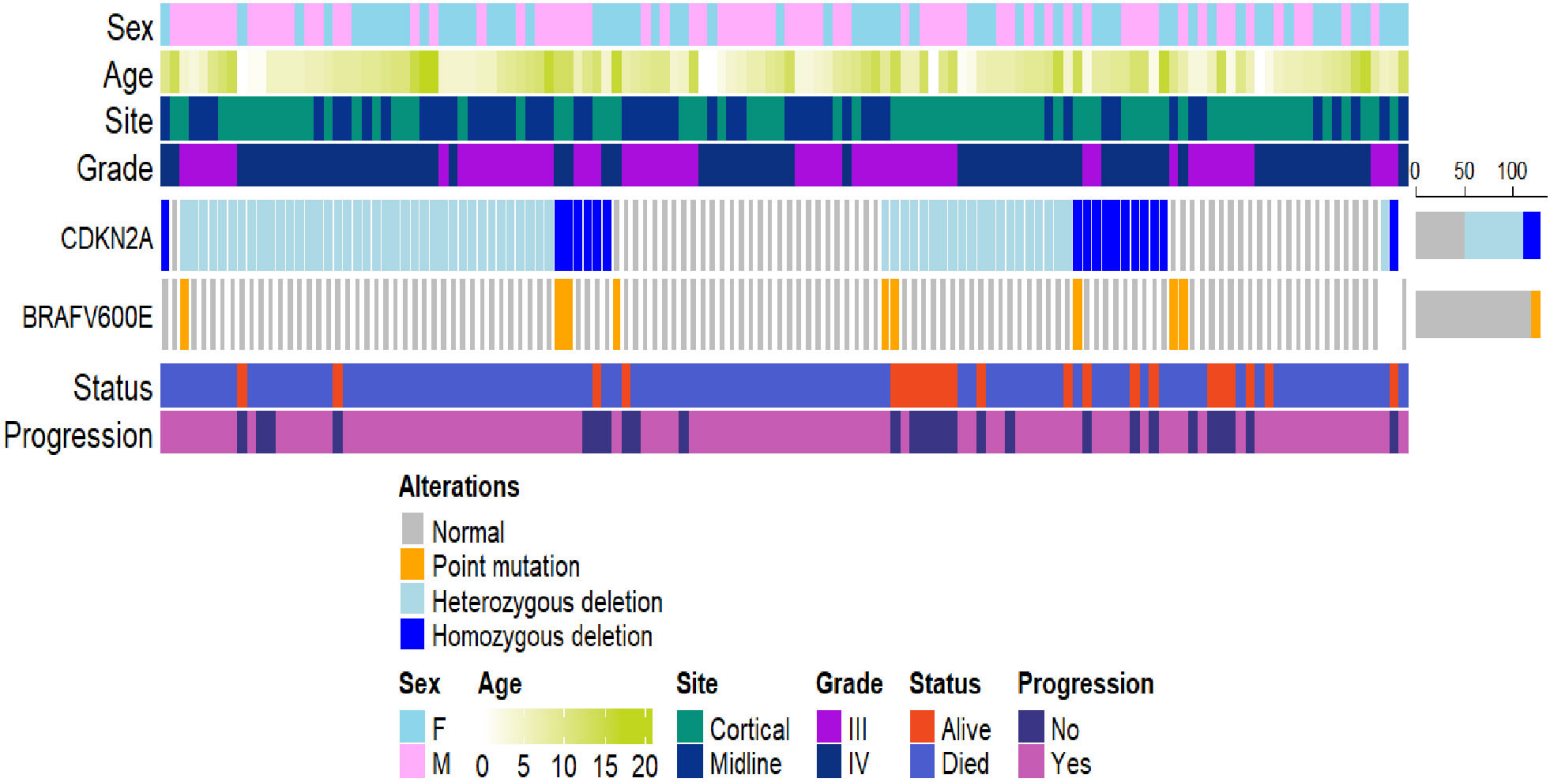
Oncoprint reporting CDKN2A and BRAF status together with key clinical features.

Patients with BRAF V600E mutation and CDKN2A deletion had a three-year PFS rate of 16.7% (95% CI: 2.8%–99.7%) (**Figure 4**). Patients with neither V600E mutation nor CDKN2A deletion had a three-year PFS rate of 17.4% (95% CI: 9.3%–32.7%). Patients with isolated BRAF V600E mutation had a three-year PFS rate of 0%. Those with isolated CDKN2A deletion showed a three-year PFS rate of 28.9% (95% CI: 19.7%–42.2%). An oncoprint illustrating the correlation of clinical data with CDKN2A and BRAF status is shown in **Figure 5**.

### Causes of death

A total of 101 patients died due to disease progression at different stages of treatment, two patients died of secondary myeloid leukemia, and one died of non-Hodgkin lymphoma.

## Discussion

This study sought to explore the prevalence of BRAF V600E mutation and CDKN2A deletion in pediatric patients with high-grade glioma (HGG) and aimed to add to the scarce data in the literature about the prognosis of this subset of patients treated with traditional surgery, radiation and chemotherapy. Although the poor prognostic impact of BRAF V600E and CDKN2A deletion is well established in LGG, their impact on prognosis in pHGG is still unclear (8).

In our study, BRAF V600E was documented in 7% of cases. Different studies have revealed a prevalence rate of BRAF V600E between 10% and 25% (9). The CDKN2A homozygous deletion rate in our cohort was 13.8%, which is consistent with previous studies that reported CDKN2A homozygous deletion in 19% of pHGG patients (12).

Our results showed that BRAF V600E mutation, alone or in combination with CDKN2A homozygous deletion, did not have a significant impact on PFS in pediatric patients with high-grade glioma. This might in part be due to the lower rate of BRAF V600E patients in our population. This result is similar to a study by Frazao et al, which showed no statistically significant correlation when analyzing progression-free survival (PFS) differences between BRAF wild type and BRAF V600E mutated tumors (20).

Our data also showed that CDKN2A status had no significant impact on progression-free survival, although patients with homozygous deletion trended to a longer PFS than those who were wild type. This data is consistent with a study by Mackay et al. who reported the molecular data of 1000 pediatric HGG which showed that homozygous CDKN2A/B deletion has been identified as a favorable prognostic factor in pHGG. This finding was somewhat subtype dependent, with PXA-like tumors having a worse prognosis and may suggest that both the mutation profile of the tumor, as well as its histologic subtype, can impact the role of homozygous CDNK2A deletion in prognosis. Diffuse intrinsic pontine gliomas, which have the worst prognosis of the pHGG, rarely have deletions in CDKN2A (14). The ability to understand the relevance of the improved outcome of the patients with homozygous deletion of CDKN2A would require a more comprehensive molecular analysis of these tumors and while the resources for this study were limited, such an analysis could take place in the future in collaboration with other institutions looking at this patient population.

In our study, age did not detect a significant impact on survival with the 3-year PFS for patients aged less than 3 of (50%) compared to those three years and above (20%), likely due to the small number in the younger age cohort. The prognosis of high-grade glioma is generally negatively correlated with increasing age. Infantile HGG has a reported 5-year survival rate of 64.1% in patients under the age of one (21). This likely reflects the different driver mutations (MET, ROS1, NTRK1,2,3, and ALK) in this patient population (5).

Tumor location played a significant role in patient outcome with cortical tumors and midline tumors having a 3-year PFS of 33.5% and 8.9%, respectively, mostly related to the feasibility of resection of cortical tumors compared to midline tumors. Eisenstat et al. reported children with midline HGG having a 5-year OS of 25% compared to other locations with an OS of 36% (22).

In our study, gross total resection was associated with better 3-year PFS than STR (42.1% vs. 10.5%, respectively). This data matched with results from a meta-analysis of 37 studies involving 1387 pediatric patients with high-grade gliomas, which reported that gross total resection was independently associated with better overall survival compared with subtotal resection and biopsy (23). The European protocol HIT-GBM-C reported that the degree of surgical excision correlated with the effectiveness of radiotherapy and chemotherapy (24).

The 3-year PFS and OS of the whole cohort were 23.4% and 26.2% respectively. These results are consistent with previous reports of 30% 5-year overall survival for children with HGG (25).

In conclusion, this retrospective study provides valuable insights into the prevalence and prognostic impact of BRAF V600E mutation and CDKN2A deletion in pediatric high-grade glioma. The findings suggest that tumor site and extent of surgical excision are important prognostic factors in HGG. However, the presence of BRAF V600E mutation or CDKN2A deletion did not significantly impact progression-free survival in this cohort. Further studies with larger sample sizes, especially related to patients with BRAF V600E mutations, more comprehensive genomic and methylation -based profiling, and longer follow-up periods are needed to extend these findings and explore other potential prognostic markers in pediatric high-grade glioma.

## Data Availability

The datasets presented in this study can be found in online repositories. The names of the repository/repositories and accession number(s) can be found in the article/**Supplementary Material**.
