# Supplementary material for: Prevalence and prognostic impact of BRAF V600E mutation and CDKN2A deletion in pediatric high-grade glioma
Source: Front Oncol. 2025 Aug 8;15:1537268. doi: 10.3389/fonc.2025.1537268 (PMC12370741; doi:10.3389/fonc.2025.1537268)
Supplement: Supplementary file 1 [file SupplementaryFile1.pdf]

Supplementary figure (1) Treatment scheme for children with HGG (as per CCH-945 COG protocol)

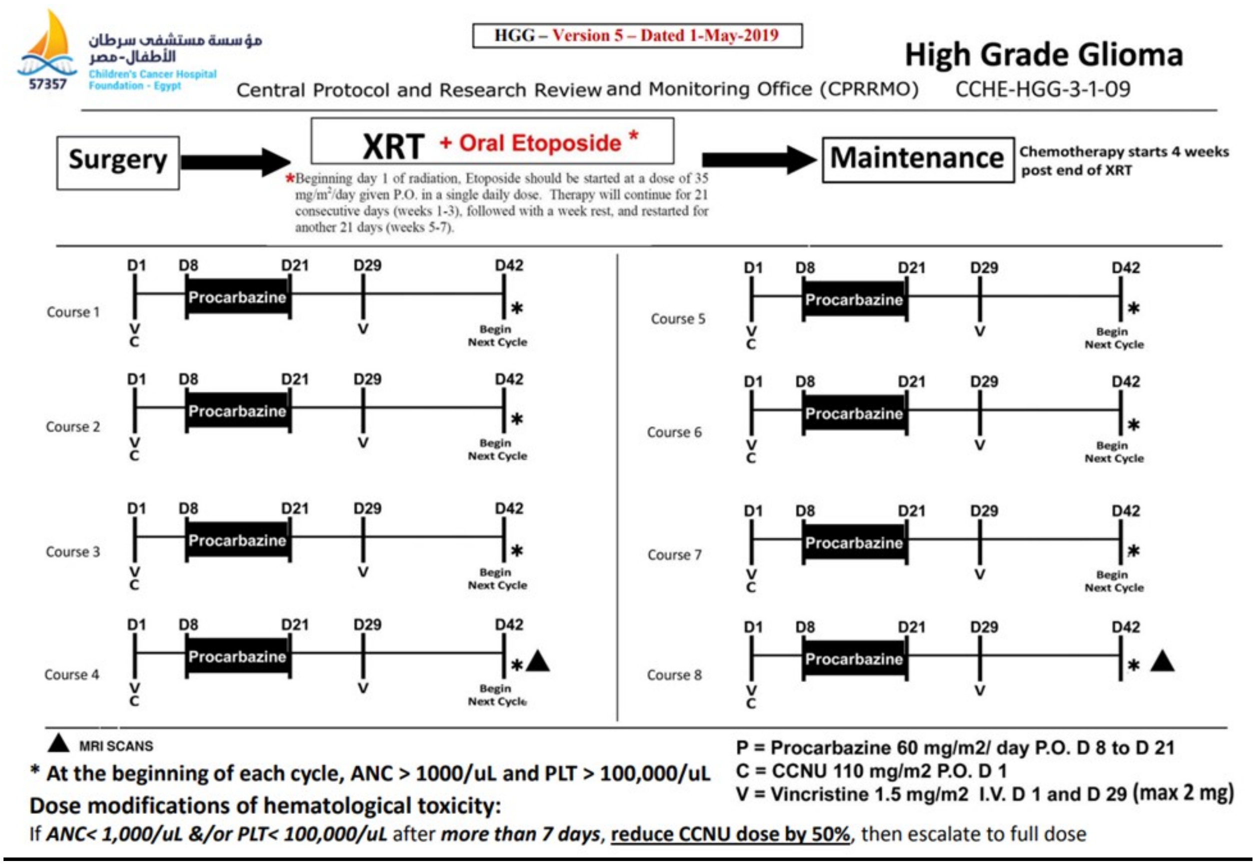

Supplementary figure (2) Treatment scheme for infants with HGG (as per Baby POG protocol)

POG #9233/34
APPENDIX I
Schema of Therapy

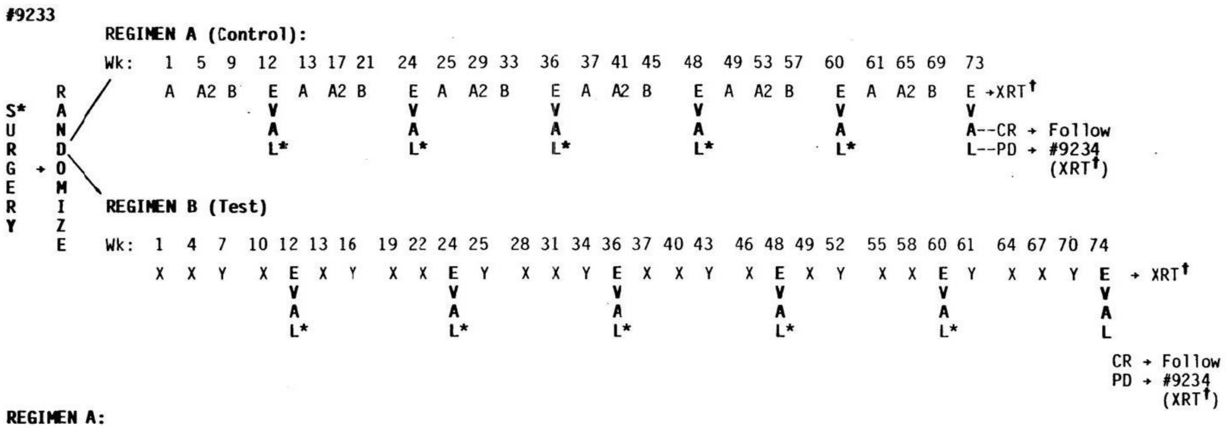

REGIMEN A:

<u>Cycle A</u>
Day 1: Vcr 0.065 mg/kg
Cyc 65 mg/kg
Mesna 13 mg/kg hrs 0, 3, 6, 9, 12
Days 8, 15, 22: Vcr 0.065 mg/kg

<u>Cycle A2</u>
Day 1: Vcr 0.065 mg/kg
Cyc 65 mg/kg
Mesna 13 mg/kg hrs 0, 3, 6, 9 12
Day 8: Vcr 0.065 mg/kg

<u>Cycle B</u>
Day 1: CDDP 4.0 mg/kg
Days 3 & 4: VP-16 6.5 mg/kg

Supplementary tables

Table (1)Criteria for response assessment incorporating MRI and clinical factors.

**Criterion**	**CR**	**PR**	**SD**	**PD**
Tl-Gd +	None	≥50% ↓	<50% ↓ to <25%↑	≥25% ↑
T2/FLAIR	Stable or ↓	Stable or ↓	Stable or ↓	↑ †
**New lesion**	**None**	None	None	Present†
Corticosteroids	**None**	Stable or ↓	Stable or ↓	NA‡
Clinical status	Stable or ↑	Stable or ↑	Stable or ↑	↓†
Requirement for response	All	All	All	**Any ‡**
Summary of HGG response criteria	Requires all of the following: complete disappearance of all enhancing measurable and non-measurable disease sustained for at least 4 weeks; no new lesions; stable or improved non-enhancing (T2/FLAIR) lesions; patients must be off corticosteroids (or on physiologic replacement doses only) and Stable or improved clinically. Note: Patients with non-measurable disease only cannot have achieved CR; the best response possible is SD	Requires **all** of the following: ≥50% decrease compared with baseline in the sum of products of perpendicular diameters of all measurable enhancing lesions sustained for at least 4 weeks; no progression of non-measurable disease; no new lesions; stable or improved 11011e1ihancing (T2/FLAIR) lesions on same or lower dose of corticosteroids compared with baseline scan; the corticosteroid dose at the time of scan evaluation should be no greater than the dose at time of baseline scan; and stable or improved clinically	Requires all of the following: Does not qualify for CR, PR or progression; stable non-enhancing (T21FLAIR) Lesions on the same or lower dose of corticosteroids compared with baseline scan. In the event that the corticosteroid dose was increased for new symptoms and signs without confirmation of disease progression on neuroimaging, and subsequent follow-up imaging shows that this increase in corticosteroids was required because of disease progression, the last scan considered to show SD will be the scan obtained when the corticosteroid dose was equivalent to the baseline dose	Defined by any of the following:≥25% increase in the sum of the products of perpendicular diameters of enhancing lesions compared with the smallest tumor measurement obtained either at baseline (if no decrease) or best response on stable or increasing doses of corticosteroids †;significant increase in T21FLAIR non-enhancing lesion on stable or increasing doses of corticosteroids compared with baseline scan or best response after initiation of therapy † not caused by comorbid events (e.g., radiation therapy, demyelination, ischemic injury, infection, seizures, postoperative changes or other treatment effects); any new lesion; clear clinical deterioration not attributable to other causes apart from the tumor (e.g., seizures, medication adverse effects, Complications of therapy, cerebrovascular events, infection, etc.) or changes in corticosteroid dose; failure to return for evaluation as a result of death or deteriorating condition or clear progression of non-measurable disease

Table (2): List of primers used in the current study

Primers	Sequence (5'-3')	Annealing temperature	Product size
**BRAF - Forward**	5’-TGCTTGCTCTGATAGGAAAAT-3’	**53°C**	**222bp**
**BRAF - Reverse**	5’-TCAGGGCCAAAAATTTAATCA-3’		

<u>Table (3): PCR program for BRAF gene</u>

**Step**	**Temperature**	**Time**	**Cycle number**
**Denaturation**	95°C	10 minutes	1
**Amplification for *BRAF* gene**	95°C	1 minute	40
	53°C	1 minute	
	72°C	1 minute	
**Elongation**	72°C	10 minutes	1
**Cool and hold**	4°C	∞	1
**Step**	**Temperature**	**Time**	**Cycle number**
**Denaturation**	95°C	10 minutes	1
**Amplification for *BRAF* gene**	95°C	1 minute	40
	53°C	1 minute	
	72°C	1 minute	
**Elongation**	72°C	10 minutes	1
**Cool and hold**	4°C	∞	1

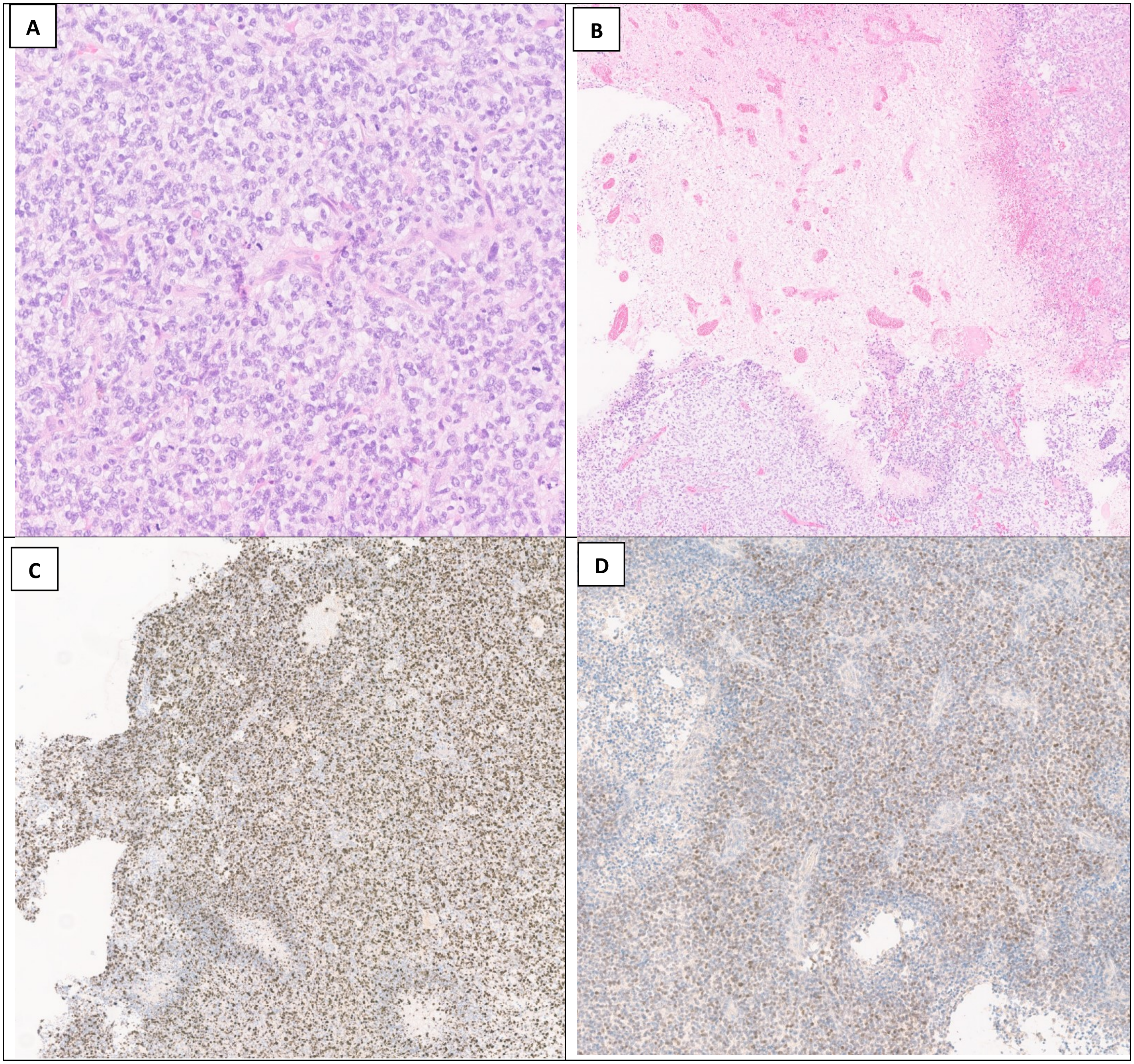

Figure (3): High grade glioma compatible with the diagnosis of Diffuse high grade glioma, WHO grade 4. **(A)** Moderately cellular neoplasm with frequent mitosis, H&E stain X20. **(B)** Palisaded necrosis is evident in many areas, H&E stain X20. **(C)** Ki-67 immunostaining showing high proliferation index reaching up to 70%, Ki67 IHC X20. **(D)** P53 shows moderate to strong nuclear reaction in 60% of tumor cells compatible with mutant pattern of expression, P53 IHC X10.

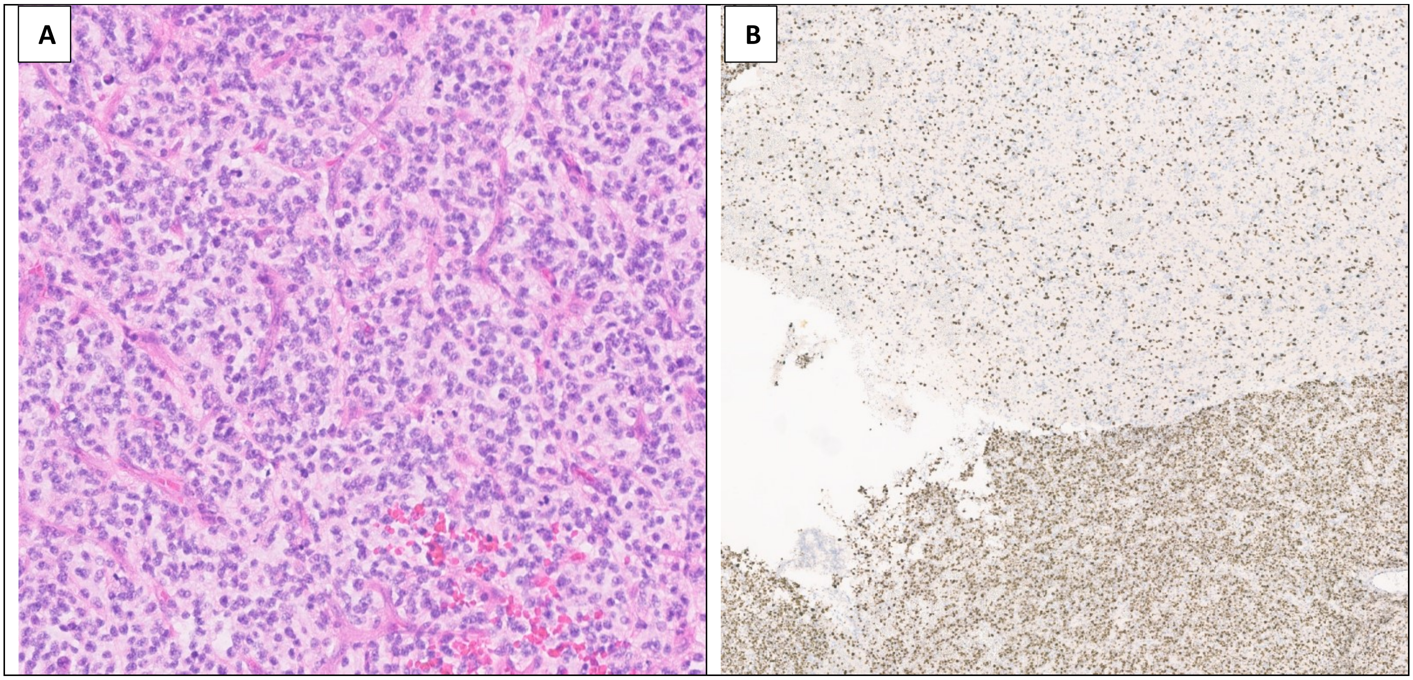

Figure (4): High grade glioma compatible with the diagnosis of Astrocytoma WHO grade 3. **(A)** Moderately cellular neoplasm with scattered mitosis, H&E stain X20. **(B)** Ki-67 immunostaining showing high proliferation index in cellular areas reaching up to 70%, Ki67 IHC X20.
